# Cytotoxicity of 9,10-Phenanthrenequinone Impairs Mitotic Progression and Spindle Assembly Independent of ROS Production in HeLa Cells

**DOI:** 10.3390/toxics10060327

**Published:** 2022-06-16

**Authors:** Seul Kim, Jiyeon Leem, Jeong Su Oh, Jae-Sung Kim

**Affiliations:** 1 Division of Radiation Biomedical Research, Korea Institute of Radiological and Medical Sciences, Seoul 01812, Korea; sseul0519@naver.com; 2 Department of Integrative Biotechnology, College of Biotechnology and Bioengineering, Sungkyunkwan University, Suwon 16419, Korea; limjiyeon0619@gmail.com

**Keywords:** 9,10-phenanthrenanquinone, reactive oxidative stress, spindle assembly, HeLa cells, mitosis

## Abstract

The polycyclic aromatic hydrocarbon quinone derivative 9,10-phenanthrenequinone (9,10-PQ) is one of the most abundant and toxic components found in diesel exhaust particles (DEPs). These DEPs are created during diesel fuel combustion and are considered the main source of urban air pollution. As 9,10-PQ can produce excessive reactive oxygen species (ROS) through redox cycling, it has been shown to exert potent cytotoxic effects against various cell types. However, the mechanisms underlying this cytotoxicity remain unclear. In this study, we showed that 9,10-PQ exerts cytotoxicity by impairing mitotic progression and spindle assembly in HeLa cells. Exposure to 9,10-PQ impaired spindle assembly and chromosome alignment, resulting in delayed mitotic entry and progression in HeLa cells. Furthermore, 9,10-PQ exposure decreased the CEP192 and p-Aurora A levels at the spindle poles. Notably, these mitotic defects induced by 9,10-PQ were not rescued by scavenging ROS, implying the ROS-independent activity of 9,10-PQ. Therefore, our results provide the first evidence that 9,10-PQ exerts its cytotoxicity through specific inhibition of mitotic progression and spindle assembly, independent of ROS.

## 1. Introduction

Diesel exhaust particles (DEPs) generated by diesel fuel combustion are considered to be one of the most toxic air pollutants [1,2]. Diesel exhaust particles are toxic mixtures of particulates composed of carbon surrounded by organic components such as polycyclic aromatic hydrocarbons, nitroaromatic hydrocarbons, heterocycles, aldehydes, and quinones [3,4]. In general, DEPs range in size from 100 to 200 nm and thus can easily reach deep into the lungs upon inhalation [5,6]. Moreover, the small size of DEPs makes them highly penetrable, thus having the potential to translocate to the bloodstream and secondarily reach other organs [7].

One of the major and most toxic particles found in DEPs and particulate matter (PM) is the polycyclic aromatic hydrocarbon quinone 9,10-phenanthrenequinone (9,10-PQ) [8,9]. Because of its electrophilic characteristics, 9,10-PQ has been shown to generate excessive reactive oxygen species (ROS) via redox cycling [10], which impairs a wide range of biochemical pathways, leading to a variety of pathological consequences [11]. It is well-known that excessive ROS exposure results in oxidative damage to nucleic acids and elicits proteotoxic stress, which in turn induces apoptosis and cell death [12,13]. Consistent with the adverse effects of ROS, 9,10-PQ has been shown to exert cytotoxic activity in many biological systems [14]. For instance, it has been well-established that DEP exposure increases the risk of developing respiratory and cardiovascular diseases [15]. Moreover, 9,10-PQ exerts toxic effects on human skin cells [16,17,18]. In addition, 9,10-PQ has been suggested to elicit potent toxicity in the vascular and nervous systems and to exacerbate the pathogenesis of neurodegenerative diseases [19,20]. Therefore, 9,10-PQ-induced cytotoxicity in various cells is considered to be the result of harmful ROS production. However, the chemical properties of 9,10-PQ remain unclear.

Mitosis is a part of the cell cycle by which a cell replicates its chromosomes and then segregates them, producing two identical nuclei in preparation for cell division [21]. Once mitosis fails to proceed under exposure to cytotoxic compounds, it often leads to mitotic cell death [22,23,24]. It is well-known that animal cells have mechanisms to avoid cell cycle progression failure, called Gap 1 (G1)/ DNA synthesis (S) and Gap2 (G2)/mitosis (M) checkpoints, to survey their genomes following exposure to cytotoxic compounds before transitioning to the next phase [21]. Cyclin-dependent kinase (Cdk)-cyclin complexes are general cell cycle regulators and M-phase promoting factors (MPFs) [25]. Among the Cdk-cyclin complexes, the Cdk1-cyclin B1 complex is a major regulator of the G2/M transition, and Cdk1 phosphorylation initiates mitosis [25,26]. Cells also monitor chromosome separation during mitosis through the spindle assembly checkpoint (SAC), which is important for kinetochore-microtubule (kMT) attachment for the correct segregation of two sister chromatids [27]. In addition, severely prolonged mitosis can lead to cell death, as determined by apoptosis regulators [28,29,30]. Mitotic spindles play essential roles in aligning chromosomes to ensure that each daughter cell receives a genome with conserved integrity. Furthermore, the spindle microtubules are nucleated from centrosomes, which are the major microtubule-organizing centers and contain centrioles [31]. CEP192, a crucial regulator of mitosis, recruits Aurora A to the centrosome for microtubule nucleation and centrosome separation and promotes sequential activation such as Aurora A phosphorylation for correct bipolar spindle assembly [32,33]. CEP192 and phosphorylation of Aurora A are essential for mitotic progression and spindle assembly [32,34]. Thus, mitosis is a critical step in maintaining cell proliferation, and proper regulation of spindle and centrosome assembly during mitosis is important for cell survival following exposure to cytotoxic compounds [21,26,35,36,37].

Although the cytotoxicity of 9,10-PQ associated with ROS has been well-established, the effect of 9,10-PQ on mitotic progression has not been explored. In this study, we evaluated the effect of acute exposure to 9,10-PQ on mitotic progression in human somatic cells in vitro. We found that 9,10-PQ exposure disturbed mitotic progression by impairing spindle assembly and chromosome alignment in HeLa cells. Moreover, mitotic defects induced by 9,10-PQ were not rescued despite ROS scavenging, implying that 9,10-PQ exerts cytotoxicity during mitotic progression in an ROS-independent manner.

## 2. Materials and Methods

### 2.1. Cell Culture and Treatment

HeLa cells were purchased from the American Type Culture Collection (CCL-2; ATCC; Manassas, VA, USA). The cells were cultured in Dulbecco’s modified Eagle’s medium (DMEM; Corning, NY, USA) and were supplemented with 10% fetal bovine serum (FBS; Corning) and 1% penicillin/streptomycin (GeneDepot; Barker, TX, USA). Cells were incubated in a humidified atmosphere containing 5% CO_2_ at 37 °C. For cell cycle synchronization, 4 × 10^5^ cells were seeded in a 60 mm cell culture dish (Corning), and a double-thymidine (Thy-Thy) block was performed as previously described [38]. For chemical treatment, 2 × 10^5^ cells were seeded in each well of a 6-well plate or 35 mm confocal dish (SPL; Naechon, Pocheon,, Korea), to which was added a solution of 9,10-PQ (275034-25G, Sigma, Burlington, MA, USA) in dimethyl sulfoxide (DMSO) diluted in the medium to a concentration of 2.5 µM during incubation. Control cells were treated with an equivalent volume of DMSO. To eliminate ROS, 1 mM N-acetyl-L-cysteine (NAC; A8199) was added to the medium containing 2.5 µM 9,10-PQ, as previously described [39].

### 2.2. Immunofluorescence

HeLa cells were fixed in 4% paraformaldehyde for 10 min and permeabilized in phosphate-buffered saline (PBS) with 0.1% Triton X-100 and 0.01% Tween 20. After permeabilization, the cells were blocked with 3% Bovine Serum Albumin (BSA) in PBS for 1–2 h at room temperature. HeLa cells were incubated with anti-α-tubulin antibody (1:1000, T7541; 1:100, rabbit, #ab18251, Abcam, Cambridge, MA, USA), CENP-A (1:100, #ab13939, Abcam), anti-p-Aurora A (1:250, 3079, Cell Signaling Technology, Danvers, MA, USA), or anti-CEP192 antibody (AR07-PA0001, Young In Frontier, 1:200) at room temperature for 45 min. After washing, the cells were incubated with the secondary antibodies for 1 h at room temperature. These were Alexa Fluor 488-conjugated (A-11001, Invitrogen, 1:100) or Alexa Fluor 594-conjugated secondary antibodies (A-11020, Invitrogen, 1:100, 1:100). The chromosomes were counterstained with Hoechst. The stained cells were observed using an IN Cell Analyzer 2200 Imaging System (Cytiva, MA, USA) with an automated spherical aberration collar (ASAC) 40 × lens and a confocal laser-scanning microscope (LSM 880; Zeiss, Germany). ZEN LSM software (Zeiss, Germany) was used to measure and analyze the fluorescence intensity. For an analysis of kMT attachment, HeLa cells were incubated in ice-cold medium for 10 min. After cold treatment, the cells were fixed and subjected to immunofluorescence analysis.

### 2.3. Immunoblotting

The cells were harvested and lysed using RIPA lysis and extraction buffer (GeneDepot, Katy, TX, USA), 1% phosphatase inhibitor (GeneDepot) and 1% protease inhibitor cocktail (GeneDepot) on ice for 10 min, and debris was removed by centrifugation. The proteins were separated using 8% SDS-PAGE and transferred onto polyvinylidene difluoride membranes (Amersham^TM^ Hybond^TM^, GE Healthcare, Chicago, IL, USA). The membranes were blocked in TBS-T containing 3% BSA for 1 h at room temperature and then incubated with primary antibodies against β-actin (1:5000, 4967S, Cell Signaling Technology), cyclin B1 (1:1000, 4138, Cell Signaling Technology), p-MPM2 (1:1000, 05-368, e, Sigma, Burlington, MA, USA), pT288-Aurora A (1:1000, 3079S, Cell Signaling Technology, Danvers, MA, USA), cleaved-PARP (1:1000, 9541S Cell Signaling Technology), or pY15-Cdk1 (1:1000, 9111, Cell Signaling Technology) overnight at 4 °C. After washing thrice, the membranes were incubated with the appropriate HRP-conjugated anti-rabbit (1:2500, GeneDepot) or anti-mouse (1:2500, GeneDepot) secondary antibodies for 30 min at room temperature. The HRP-conjugated secondary antibody was detected using an enhanced chemiluminescence detection system (Amersham Life Science, Piscataway, NJ, USA), and the bands were imaged using an Amersham Imager 600 system (GE Healthcare).

### 2.4. Cell Viability Assay

A total of 1.5 × 10^4^ HeLa cells were seeded into each well of a 96-well plate and incubated at 37 °C overnight. Next, solutions of 0.125, 0.25, 0.5, 1, 2, 4, 8, and 10 µM of 9,10-PQ were diluted in the medium. After 24 h treatment, the medium was aspirated and 100 µL of a 10% cell viability assay kit solution (Cyto X^TM^; LPS solution, Daejeon, Korea) was added to each well. The samples were then incubated at 37 °C for an additional 1 h in a 5% CO_2_ incubator. Next, the optical density (OD) was measured at 460 nm in a microplate reader (Molecular Devices, San Jose, CA, USA).

### 2.5. Statistical Analyses

Statistical analyses were performed using GraphPad Prism version 9.0.0 for windows (GraphPad Software, La Jolla, CA, USA). The results are representative of at least three independent experiments unless otherwise specified, and each experimental group included at least 25 mitotic cells. The significance of differences between two groups was analyzed using Student’s *t*-test, and comparisons between more than two groups were analyzed using one-way ANOVA with Tukey’s post-hoc test; *p*-values < 0.05 were considered statistically significant.

## 3. Results

### 3.1. Exposure to 9,10-PQ Disturbs Mitotic Progression

**Figure 1:**
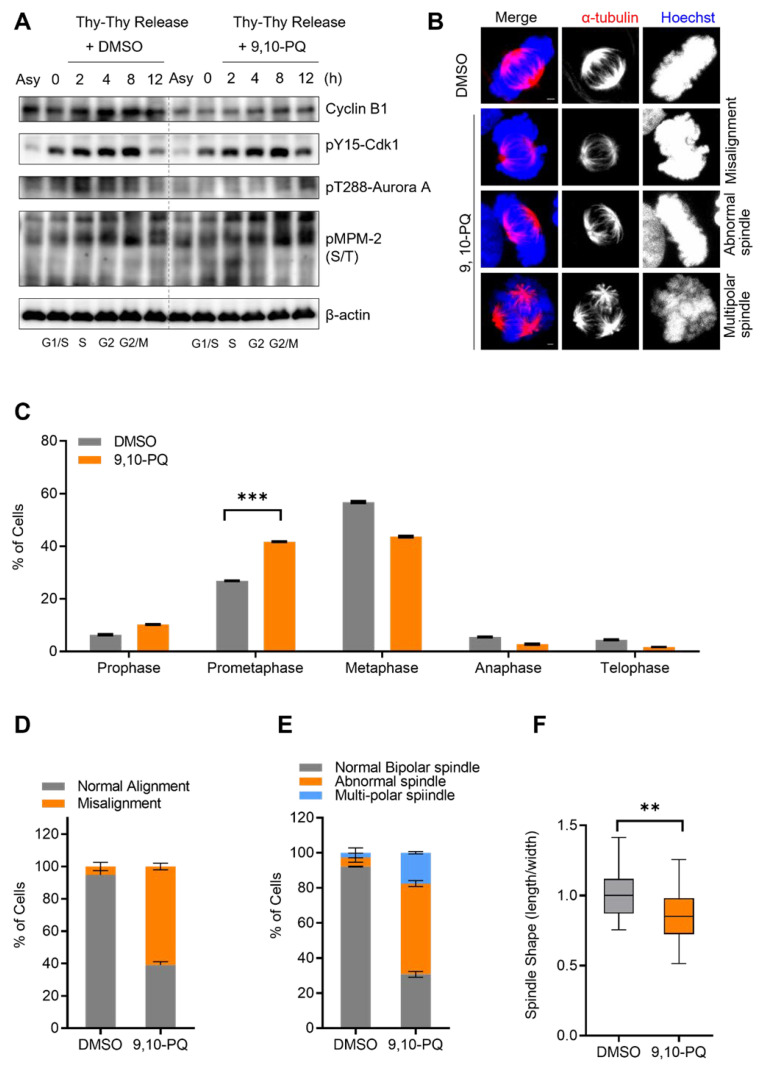
9,10-PQ disturbs normal mitotic progression. (**A**) HeLa cells synchronized at the G1/S stage by double thymidine treatment cultured with 2.5 µM 9,10-PQ after replacement of the medium. Cells were harvested at the indicated times and analyzed by immunoblotting with cyclin B1, pY15-Cdk1, p-Aurora A, p-MPM-2, and β-actin antibodies. Antibody of p-MPM-2 recognizes a variety of phosphorylated proteins during mitosis. Asynchronized cells (Asy) were not arrested by thymidine. (**B**–**F**) HeLa cells incubated with DMSO or 9,10-PQ and stained with the indicated antibodies. Spindles and chromosomes were visualized by α-tubulin and Hoechst stain, respectively. (**B**) Representative images of HeLa cells showing chromosome and spindle configuration during metaphase. Scale bar, 5 μm. (**C**) Quantification of mitotic cells. (**D**) The percentage of metaphase cells with misaligned chromosomes among the total metaphase cells. (**E**) Quantification of spindle abnormalities during mitosis. (**F**) Aspect ratio of spindle length to width per metaphase cell. Data are shown as mean ± SEM from at least three independent experiments (*n* = 25 miotic cells for each quantification and group). *** *p* < 0.0001, ** *p* < 0.001; ns, not significant.

Based on previous reports, DEPs induce genotoxicity in normal human embryonic lung cells, and its potential inhibitory effects on cell cycle progression through RNA expression have been noted [40,41]. First, to confirm the cytotoxicity of 9,10-PQ in HeLa cells, we treated the cells with 1, 2, 4, or 8 µM 9,10-PQ for up to 24 h, in agreement with the methods of a previous study [16], and examined the cell morphology, viability, and protein expression (Figure S1A–C). Our results revealed that the cells adopted a rounded appearance after exposure to 2 and 4 µM 9,10-PQ and that most cells were dead following treatment with 8 and 10 µM 9,10-PQ (Figure S1A). Consistent with these morphological observations, cell viability significantly decreased after exposure to 8 and 10 µM 9,10-PQ (Figure S1B), and cleaved-PARP expression increased (Figure S1C). Next, a Western blot analysis revealed the accumulation of cyclin B1, which indicates cell cycle arrest in the G2/M phase (Figure S1C). Thus, we hypothesize that exposure to 2 and 4 µM of 9,10-PQ inhibits mitotic entry and arrests the cell cycle at the mitotic phase. To further determine the potential mitosis-disrupting effects of 9,10-PQ, HeLa cells were synchronized at the G1/S phase using double thymidine and were released into fresh media. The Western blot analysis after the release from double thymidine arrest revealed that 9,10-PQ inhibited the accumulation of cyclin B1, implying a delay in the G2/M transition during cell cycle progression (Figure 1A). Consistent with cyclin B1 levels, the expressions of the mitotic indicators pY15-Cdk1, p-Aurora A, and p-MPM-2 were delayed by 9,10-PQ exposure (Figure 1A), implying that mitotic entry was prolonged. To further investigate the effect of 9,10-PQ exposure on mitotic progression, we examined mitotic chromosomes and spindles in metaphase using immunofluorescence microscopy. Specifically, after exposure to 9,10-PQ, the percentage of prometaphase cells accumulated, displaying various abnormalities in spindle and chromosome organization (Figure 1B–F), such as chromosome displacement (Figure 1D) and spindle morphological abnormalities (Figure 1E,F). Conversely, negative control cells (DMSO) showed regular spindle morphology with well-aligned chromosomes. Thus, our results suggest that 9,10-PQ disrupts normal mitotic progression.

### 3.2. 9,10-PQ Exposure Impairs kMT Attachment during Mitosis

**Figure 2:**
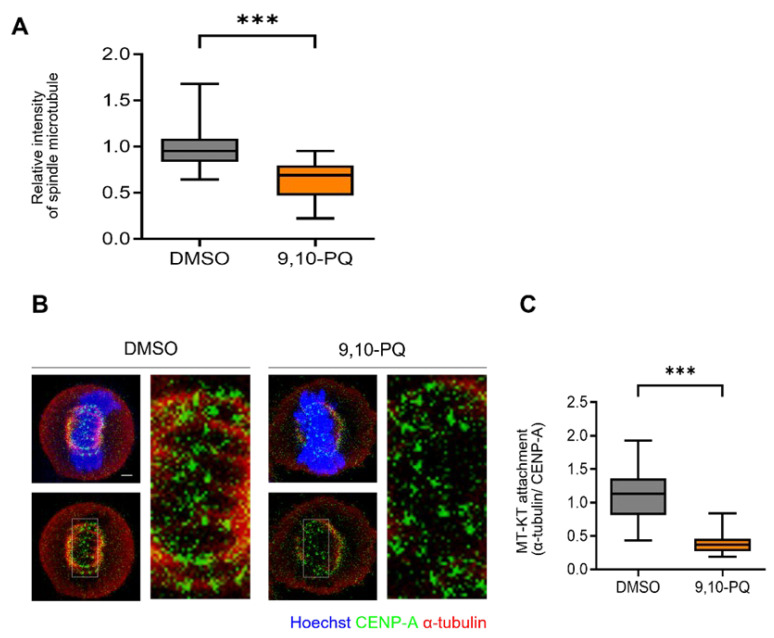
9,10-PQ inhibits spindle assembly during mitosis. (**A**) Metaphase HeLa cells incubated with DMSO or 9,10-PQ and stained with α-tubulin and Hoechst, respectively, for the quantification of spindle microtubule intensity. Data are shown as the mean ± SEM from at least three independent experiments (*n* = 100 mitotic cells for each quantification and group). *** *p* < 0.0001; ns, not significant. (**B**) Representative images of metaphase HeLa cells incubated with DMSO or 9,10-PQ and stained with CENP-A, α-tubulin, or Hoechst after cold treatment. Scale bar, 5 μm. The spindles and chromosomes were visualized using α-tubulin and Hoechst stain, respectively. (**C**) Aspect ratio of tubulin intensity to CENP-A intensity at kinetochores.

It is well-established that mitotic spindle dynamics and centrosome maturation regulate chromosome organization and mitotic progress in human cells [33,42]. Therefore, we investigated the integrity of the spindle microtubules after 9,10-PQ exposure. Consistent with the impairment of spindle assembly, the relative intensity of α-tubulin significantly decreased in cells exposed to 9,10-PQ (Figure 2A). To further assess the effect of 9,10-PQ exposure on spindle assembly, we explored the stability of kMT attachments in mitotic cells exposed to 9,10-PQ. While stable kMT attachments were predominantly observed in control cells, the percentage of unattached kinetochores significantly increased in mitotic cells after 9,10-PQ exposure (Figure 2B). Indeed, 9,10-PQ exposure significantly reduced the intensity of microtubules in the vicinity of kinetochores (Figure 2C). Thus, our results suggest that 9,10-PQ exposure impairs spindle assembly and subsequent kMT attachment during mitosis, resulting in a mitotic delay.

### 3.3. 9,10-PQ Exposure Decreases Centrosome Integrity and Spindle Assembly during Mitosis

**Figure 3:**
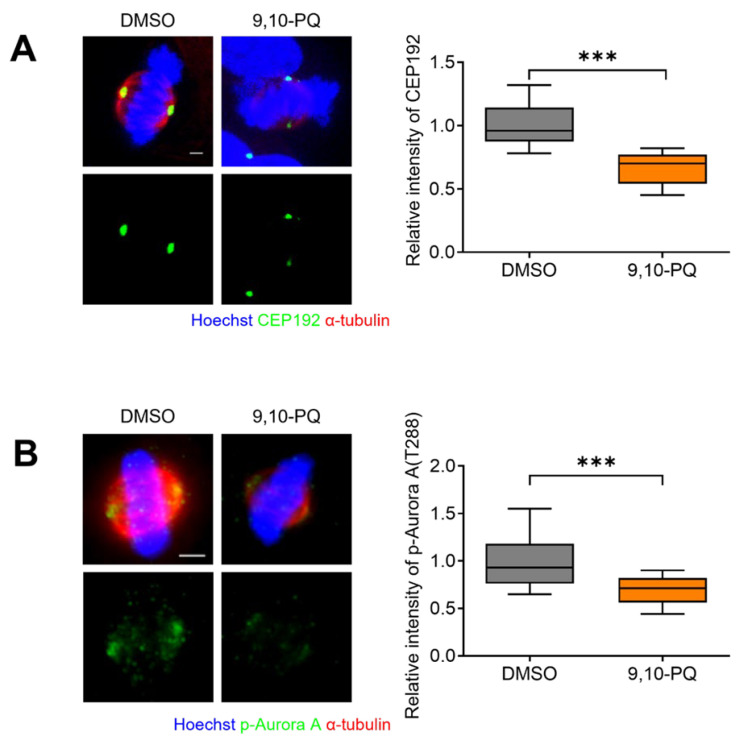
9,10-PQ interferes with the recruitment of CEP192 and p-Aurora A in centrosomes. (**A**) HeLa cells incubated with DMSO or 9,10-PQ and stained with the indicated antibodies for the quantification of CEP192 fluorescence intensities. The spindles and chromosomes were visualized using α-tubulin and Hoechst stain, respectively. Scale bar, 5 μm. (**B**) HeLa cells incubated with DMSO or 9,10-PQ and stained with the indicated antibodies for the quantification of p-Aurora A fluorescence intensity. The spindles and chromosomes were visualized using α-tubulin and Hoechst stain, respectively. Scale bar, 5 μm. Data are shown as the mean ± SEM from at least three independent experiments (*n* = 25 mitotic cells for each quantification and group). *** *p* < 0.0001; ns, not significant.

Given that 9,10-PQ induces misaligned chromosomes and abnormal spindle formation (Figure 1 and Figure 2), we hypothesized that 9,10-PQ disrupts the regulators of spindle microtubule (MT) attachment to kinetochores (KTs) and centrosome integrity. Since CEP192 and Aurora A are essential regulators of centrosome maturation and bipolar spindle assembly [32,33], we examined the localization of CEP192 and p-Aurora A using immunofluorescence microscopy. Our data showed that the intensities of both regulators were significantly reduced in metaphase cells after 9,10-PQ exposure (Figure 3A,B). Therefore, we concluded that 9,10-PQ exposure impairs normal bipolar spindle and centrosome assembly during mitosis.

### 3.4. Mitotic Defects Induced by 9,10-PQ Exposure Are Not Associated with ROS

**Figure 4:**
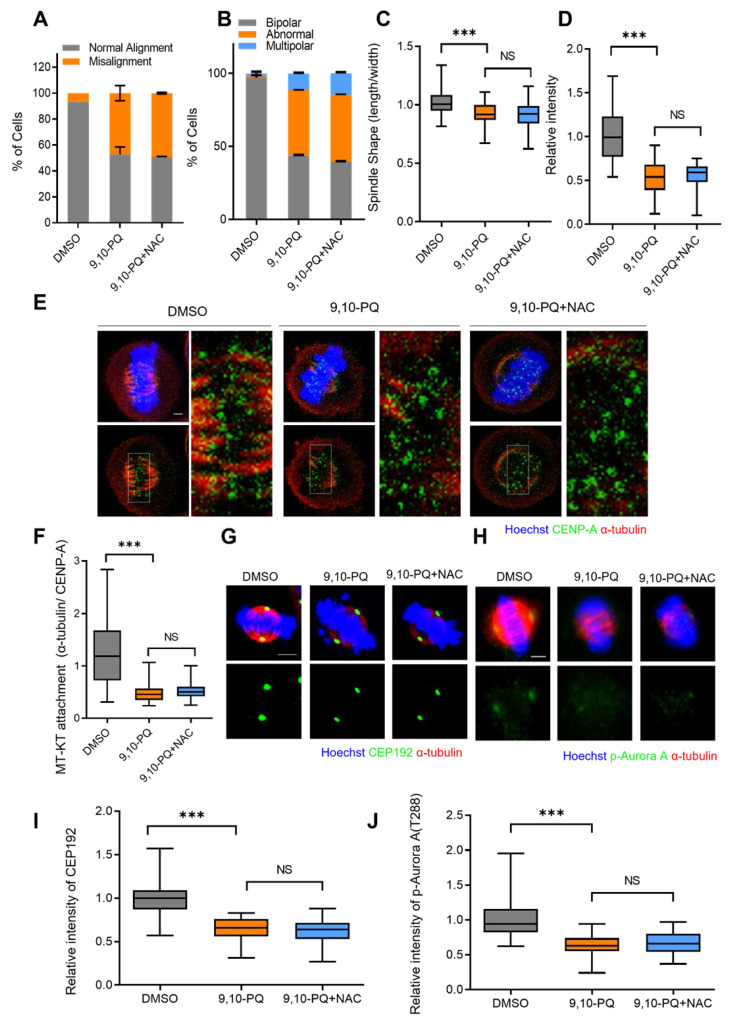
9,10-PQ impairs mitotic progression and spindle assembly in a ROS-independent manner. (**A**–**D**) HeLa cells incubated with DMSO, 9,10-PQ, or 9,10-PQ + N-acetyl-L-cysteine (NAC) and stained with α-tubulin and Hoechst, respectively. (**A**) The percentage of metaphase cells with misaligned chromosomes among the total metaphase cells. (**B**) Quantification of spindle abnormalities during mitosis. (**C**) Aspect ratio of spindle length to width per metaphase cell. (**D**) Quantification of spindle microtubule intensity of metaphase HeLa cells. Data are shown as mean ± SEM from at least three independent experiments (*n* = 100 miotic cells for each quantification and group). *** *p* < 0.0001; ns, not significant. (**E**) Representative images of metaphase HeLa cells incubated with DMSO, 9,10-PQ or 9,10-PQ + NAC and stained with CENP-A, α-tubulin, and Hoechst after cold treatment. (**F**) Aspect ratio of tubulin intensity to CENP-A intensity at kinetochores. Scale bar, 5 μm. (**G**–**J**) HeLa cells incubated with DMSO, 9,10-PQ or 9,10-PQ + NAC. Spindles and chromosomes were visualized by α-tubulin and Hoechst, respectively. Scale bar, 5 µm. (**G**) HeLa cells stained with CEP192. (**H**) HeLa cells stained with p-Aurora A. Spindles and chromosomes were visualized by α-tubulin and Hoechst, respectively. Scale bar, 5 µm. (**I**) Quantification of CEP192 fluorescence intensities. (**J**) Quantification of p-Aurora A fluorescence intensities. Data are shown as mean ± SEM from at least three independent experiments (*n* = 25 miotic cells for each quantification and group). *** *p* < 0.0001; ns, not significant.

It is well-known that 9,10-PQ can cause ROS via redox cycling. Thus, we investigated whether the mitotic defects induced by 9,10-PQ exposure were the result of excessive ROS accumulation during mitosis. To this end, we treated HeLa cells with the common ROS scavenger N-acetyl-L-cysteine (NAC) during 9,10-PQ exposure. Interestingly, mitotic defects, including chromosome misalignment, abnormal spindle formation, multipolar spindles, and kMT attachments in 9,10-PQ-exposed HeLa cells were not restored post-NAC treatment (Figure 4A–F). The low intensity of CEP192 and p-Aurora A in cells exposed to 9,10-PQ was also not rescued after NAC treatment (Figure 4G–J). Our results suggest that 9,10-PQ interferes with normal mitotic progression and impairs spindle and chromosome organization in an ROS-independent manner.

## 4. Discussion

Among the components of DEPs, one of the most potent toxicants is 9,10-PQ [8,9]. Despite the observed cellular phenomena caused by 9,10-PQ [14], its molecular mechanism of cytotoxicity in human somatic cells remains unknown. In this study, we investigated whether 9,10-PQ exposure impaired mitotic progression and noted its disruptive effects on spindle assembly and chromosome alignment in HeLa cells. Our results also indicated that exposure to 9,10-PQ disturbed the localization of CEP192 and p-Aurora A, which are essential for spindle assembly during mitosis. Therefore, our data demonstrate the first evidence of novel mechanisms for the cytotoxicity of 9,10-PQ that causes the impairment of mitotic progression, bipolar spindle assembly, and chromosome organization.

Among the cellular process that maintain cell proliferation, mitosis inherently has a high level of risk owing to the need for accurate chromosome segregation and orientation to opposite poles of the bipolar spindle for proper mitotic progression [43,44]. Spindle assembly and centrosome maturation are essential for these processes [45]. We found that exposure to 9,10-PQ inhibited mitotic entry and progression in HeLa cells. Since it is well-established that prolonged mitosis is associated with SAC activation due to insufficient spindle conditions [35,36,37], spindle assembly was impaired in 9,10-PQ-exposed cells. In fact, our data showed that exposure to 9,10-PQ disturbed bipolar spindle assembly and kMT attachment, resulting in mitotic delay and misaligned chromosomes in HeLa cells. Recently, potential antitumor agents have been reported for candidate drug repurposing that impair mitotic progression, spindle assembly, and chromosome organization in various cancer cells [46,47,48]. Given our data, it is also possible that 9,10-PQ may provide a novel antitumor agent, although selectivity on cancer cells remains to be investigated.

9,10-PQ produces ROS through redox cycling in human cells [10,14]. ROS production is thought to be related to the cell cycle; however, this connection is conflicting [49]. It has been reported that mitotic arrest elevates oxidative stress, while other sources state that oxidative stress induces mitotic arrest [49,50,51,52,53,54]. Recently, it has been reported that ROS peaks in mitosis, resulting in mitotic arrest, and that extended mitotic arrest further increases the levels of ROS [49]. Therefore, it is possible that cytotoxicity induced by 9,10-PQ is indirectly associated with cell cycle progression mediated by oxidative stress. Surprisingly, we found that antioxidant treatment with NAC could not rescue the impaired mitotic progression and spindle assembly mediated by 9,10-PQ exposure. Therefore, this indicates that 9,10-PQ exerts ROS-independent cytotoxicity through the disruption of miosis in human cells. Furthermore, our data provide evidence to address a previously reported issue, that 9,10-PQ cytotoxicity significantly increased even though ROS was removed using NAC in human skin cell lines [16]. Thus, our data suggest that the mechanism of 9,10-PQ cytotoxicity is independent of ROS in human cells.

Given that our data showed that exposure to 9,10-PQ reduced the formation of bipolar spindles, intensity of spindle microtubules, and kMT attachment in mitotic cells, we analyzed the localization of mitotic regulators, which are essential for spindle assembly and centrosome maturation. Importantly, we identified the first evidence that exposure to 9,10-PQ interferes with the recruitment of CEP192 and p-Aurora A, resulting in a lack of MT attachment to KT in an ROS-independent manner. Moreover, the Western blot analysis showed that p-Aurora A expression decreased in cells exposed to 1–4 µM of 9,10-PQ (Figure S1C). Thus, we identified an important molecular mechanism of cytotoxicity induced by 9,10-PQ in human cells, although more detailed mechanisms need to be investigated through further studies. For instance, whether 9,10-PQ exposure disrupts spindle integrity along with microtubule regulators or motor proteins remains unknown. Spindle bipolarity is maintained by microtubule dynamics to secure complete separation of replicated chromosomes [55]. Microtubule dynamics and organization are controlled by MT-associated proteins (MAPs) and motor proteins, such as augmin, TPX1, HURP, and kinesin superfamily proteins (KIFs). Thus, it is possible that the decrease in spindle integrity after 9,10-PQ exposure results from the disruption of MAPs or motor proteins. Furthermore, since 9,10-PQ is also an air pollutant and the major entry route is inhalation, further in vivo studies on the mitotic defects induced by 9,10-PQ in pulmonary cell lines and the amount of daily absorption into lungs are needed.

## 5. Conclusions

A toxic air pollutant, 9,10-PQ, is the major quinone that constitutes DEPs and PM. Although the cytotoxic effects of 9,10-PQ have been reported in a various type of cells, the underlying mechanisms of cytotoxicity in HeLa cells were previously unclear. In the present study, we demonstrated for the first time that 9,10-PQ exposure impaired mitotic progression, bipolar spindle assembly, and chromosome organization in a ROS-independent manner in HeLa cells. These results underline that harmfulness independent of ROS production should be considered, even if it is a toxic pollutant that produces ROS. Thus, our study demonstrates new insights into the properties of 9,10-PQ and its possible mechanisms of cytotoxicity.

## Data Availability

Not applicable.

## Supplementary Materials

The following are available online at https://www.mdpi.com/article/10.3390/toxics10060327/s1, Figure S1: 9,10-PQ induced mitotic arrest and reduced viability in Hela cells. (A) HeLa cells incubated with DMSO, or 1, 2, 4, 8, or 10 µM 9,10-PQ for up to 24 h. Cell mor-phology was observed by optical microscopy (×40 magnification). Yellow arrows in-dicate rounded cells in mitosis. (B) HeLa cells were treated with 0.125, 0.250, 0.5, 1, 2, 4, 8, and 10 µM 9,10-PQ for 24 h, and a cell viability assay was conducted. Data are shown as mean ± SEM from at least three independent experiments (*n* = 9) *** *p* < 0.0001. (C) HeLa cells incubated with DMSO, or 1, 2, 4, or 8 µM 9,10-PQ for up to 24 h. The cells were harvested and analyzed by immunoblotting with cyclin B1, cleaved-PARP, p-Aurora A, and β-actin antibodies.
