# Supplementary material for: Cytotoxicity of 9,10-Phenanthrenequinone Impairs Mitotic Progression and Spindle Assembly Independent of ROS Production in HeLa Cells
Source: Toxics. 2022 Jun 16;10(6):327. doi: 10.3390/toxics10060327 (PMC9227850; doi:10.3390/toxics10060327)
Supplement: Supplementary file 1 [file toxics-10-00327-s001.zip › toxics-1752063-supplementary.pdf]

# Supplementary Materials: Cytotoxicity of 9,10-Phenanthrenequinone Impairs Mitotic Progression and Spindle Assembly Independent of ROS Production in HeLa Cells

**Seul Kim, Jiyeon Leem, Jeong Su Oh and Jae-Sung Kim**

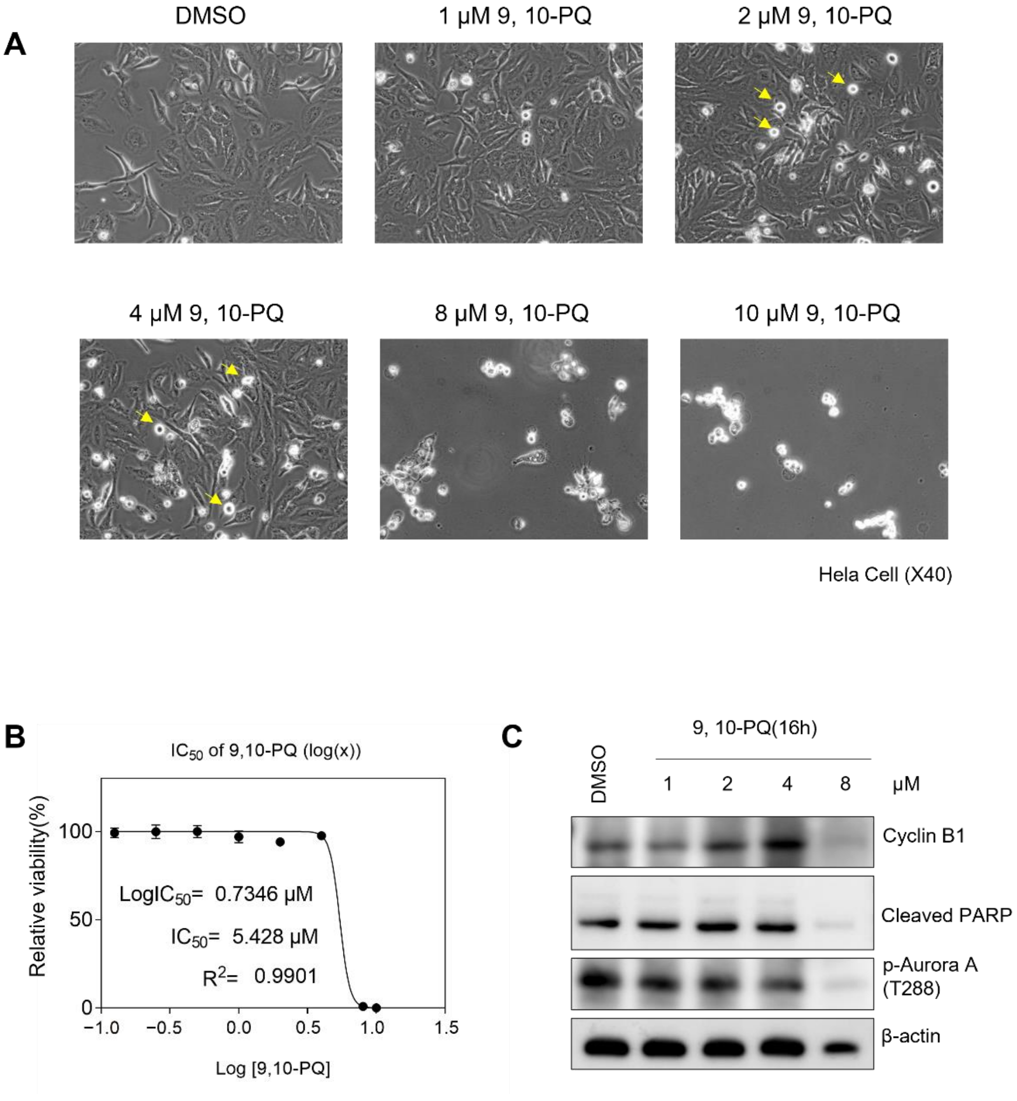


**Figure S1.** 9,10-PQ induced mitotic arrest and reduced viability in Hela cells. (**A**) HeLa cells incubated with DMSO, or 1, 2, 4, 8, or 10 μM 9,10-PQ for up to 24h. Cell morphology was observed by optical microscopy (× 40 magnification). Yellow arrows indicate rounded cells in mitosis. (**B**) HeLa cells were treated with 0.125, 0.250, 0.5, 1, 2, 4, 8, and 10 μM 9,10-PQ for 24h, and a cell viability assay was conducted. Data are shown as mean ± SEM from at least three independent experiments (n = 9) *** p<0.0001. (**C**) HeLa cells incubated with DMSO, or 1, 2, 4, or 8 μM 9,10-PQ for up to 24h. The cells were harvested and analyzed by immunoblotting with cyclin B1, cleaved-PARP, p-Aurora A, and β-actin antibodies.
